# Supplementary figures and images for: Ion concentration measurement using synthetic microfluidic papers
Source: PLoS One. 2020 Nov 19;15(11):e0242188. doi: 10.1371/journal.pone.0242188 (PMC7676646; doi:10.1371/journal.pone.0242188)

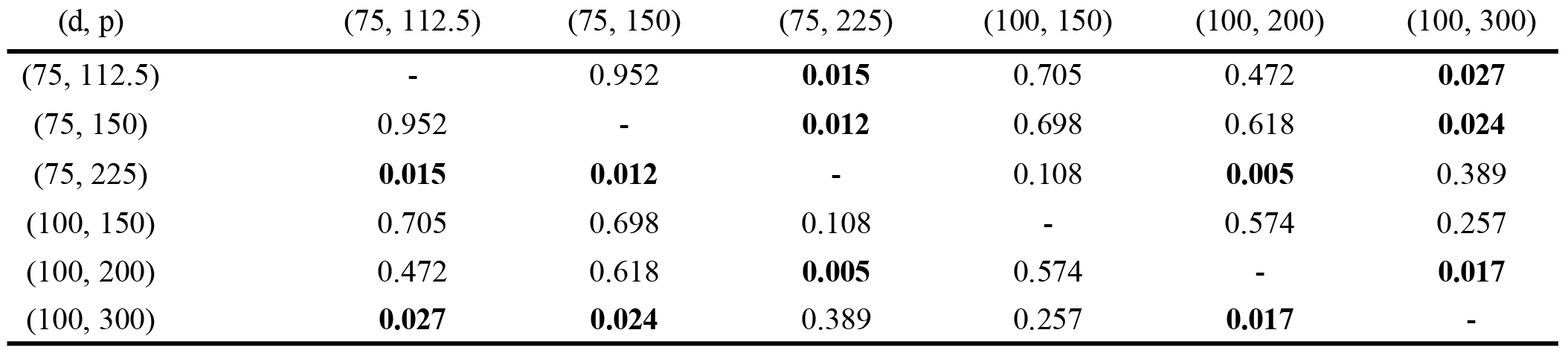

Supplement: S1 Table — (TIF) [file pone.0242188.s001.tif]

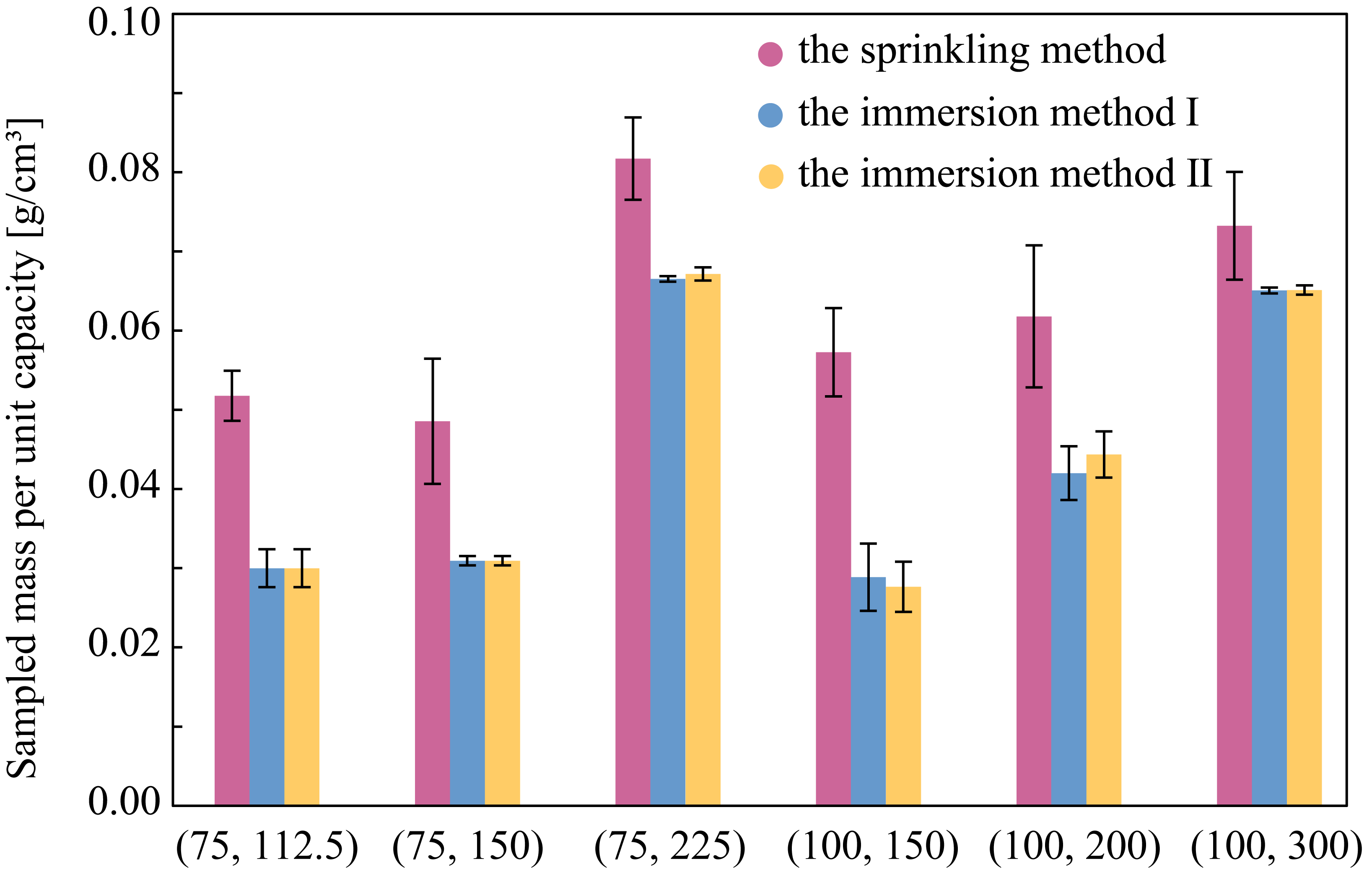

Supplement: S1 Fig — (TIF) [file pone.0242188.s002.tif]

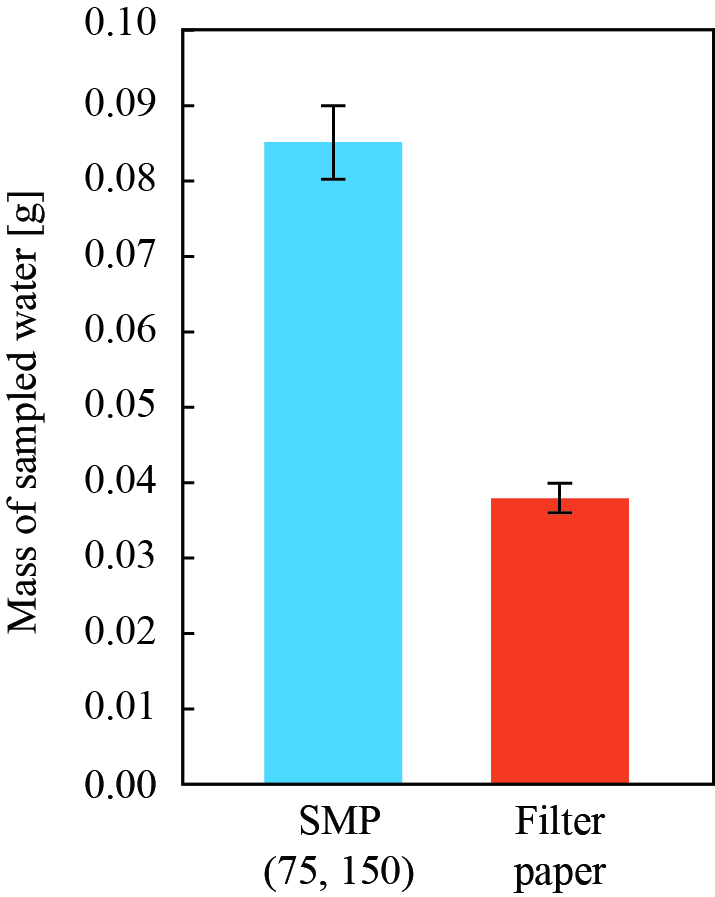

Supplement: S2 Fig — (TIF) [file pone.0242188.s003.tif]
