# Supplementary material for: Ion concentration measurement using synthetic microfluidic papers
Source: PLoS One. 2020 Nov 19;15(11):e0242188. doi: 10.1371/journal.pone.0242188 (PMC7676646; doi:10.1371/journal.pone.0242188)
Supplement: Attachment — Submitted filename: Response to Reviews_PLOSONE_2nd_Kamiya_20200928.docx [file pone.0242188.s004.docx]

**Response to Reviewers**

Our response to the two reviewers’ comments on the previously submitted manuscript for *PLOS ONE* follows. In the revised manuscripts, these comments are reflected in the main text and the electronic supporting information.

Reviewer 1

# **Comment 1**

**Please describe conditions of testing in more detail, as temperature, humidity, drying time, measurement time can all effect ion concentration measurements using microfluidic paper. The possible condition limitations are important to a medical setting.**

**Answer**

We would like to thank the reviewer for requesting essential information, which was missed. The followings are the test conditions for the measurement. For the conditions of temperature, humidity, and measurement time, detailed description was added in the line 152 of the page 6 and the line 206 of the page 8 in the revised manuscript.

Temperature: Approximately 23 ℃

Humidity: 50±5 %

Drying time: Not applicable because concentration measurement was performed immediate after sampling. We suppose sampling and measurement with the synthetic microfluidic paper are performed together as a series of operations. Thus, the discussion related to the drying time was not included in the revised manuscript.

Measurement time: within 10 seconds, result of automatic measurement process in the commercially available densitometer.

***The added sentence in the line 152 of the page 6:***

All experiments including following experiments were conducted at approximately 23℃ and humidity was kept at 50±5%.

***The added sentence in the line 206 of the page 8:***

Less than 10 seconds were taken for each measurement, which resulted from automatic measurement process of the densitometer used.

# **Comment 2**

**Please address the appropriateness of using your sample size (n=5).**

**Answer**

We consider the sample size *n* = 5 was sufficient to see variation but we conducted an extra experiment to get more convincing results, taking into account the comment of another reviewer. The previous results on precision sampling tests and sample retention tests were conducted five times with a same sampling strip. In this revision, we re-considered claims from these experimental results and regarded the results as those for the selection of suitable geometry of synthetic microfluidic paper. Then, we conducted an extra experiment to compare the synthetic microfluidic paper selected based on the prior tests with commercially available filter paper. On this experiment, we conducted liquid sampling for the two types of paper 10 times with a new paper strip every time. To avoid the variation originating from human error, sampling (that is pulling the paper strip out of liquid) was conducted with machine-controlled set-up in precision sampling tests. We believe that more appropriate data set was obtained from this experiment. Please confirm the results in newly added section from the line 250 of the page 10 in the revised manuscript.

***The added section in the line 250 of the page 10:***

**Comparison between synthetic microfluidic paper and filter paper**

Fig 9 (a) shows the coefficient of variation (CV) of sampling volume for the SMP and the filter paper, which was calculated as the ratio of the standard deviation to the mean value (N = 10). The actual volume sampled by each paper is shown in SI Fig S2. The CV value of the SMP was slightly greater than that of the filter paper. However, we performed F-test on the two normalized data set (ratio of sampled mass over its mean mass) of the SMP and the filter paper by “ftest” function in Excel, and, when an α-level of 0.05 was used, no significant difference was found between them (P = 0.382 > α). Fig 9 (b) shows the R_F. The error bars represent standard deviation. T-test performed on the two groups by “ttest” function in Excel showed no significant difference between the SMP and the filter paper (P=0.078 > α), when an α-level of 0.05 was used. On the other hand, F-test performed on the two groups showed that there is a significant difference between variation for the two samples (P = 0.0056 < α), when an α-level of 0.05 was used.

In summary of the comparison between the SMP and the filter paper, they were found to have comparable intra-assay precision in water sampling and retention, but robustness to mechanical disturbances was shown more in the SMP than the filter paper. However, there is a big difference between the SMP and the filter paper: the former was fabricated manually in a laboratory but the latter was done by mass-production process. Actually, the mass variation of the SMP and the filter paper was 0.078 and 0.033 in CV. Automated process or reduction of manual process, if possible, are expected to decrease variation of mass in SMP and consequently variation of sampling volume.

From a future perspective, sampling liquid by SMP has challenges in terms of evaluation for long-term or multi-person use. In this work, the usability of SMP for precise sampling has been confirmed in short-term period. It is also important to know long-term capability to perform precise sampling as a reference for a shelf-life. To get longer-term capability, optimization of polymer formulation for the SMP is one of the issues to be considered. Another challenge to be addressed is multi-operator tests. Depending on the application destination, it is necessary to test with a wide range of subjects who have different age and presence or absence of disease.

Reviewer 2 -Major point-

# **Comment 1**

**Overall, it is difficult to know how many replicates have been used for each experiment. Authors should clarify this point in experiments section and add n numbers in legend of each figures when applicable.**

**Answer**

We would like to thank the reviewer for this comment. The number of tests were clarified as needed.

# **Comment 2**

**In “precision sampling experiment” in experiments section, authors claimed “six samples (N=5, for each) were collected for each method…” but it is unclear. Power analysis is not provided, but I would suggest authors to perform each condition with at least 10-20 replicates.**

**Answer**

Certainly, this sentence was misleading. It means that 6 types of synthetic microfluidic paper (SMP) were prepared in different fabrication condition, and the SMP in each condition was tested five times. This detail was added from the line 138 of the page 6 in the revised manuscript.

For the number of the tests, we addressed it by focusing on one dimensional parameter of SMP to compare it with filter paper. This point is discussed in the answer for the next comment.

***The sentence in the previous manuscript:***

Six samples (N = 5, for each) were collected for each method, and sample volume was measured from the weight of the strip before and after sampling.

***The modified sentence from the line 138 of the page 6 in the revised manuscript:***

6 types of SMPs were prepared in different fabrication condition as in Table 1, and the SMP in each condition was tested using a same paper strip five times *(n* = 5); sample volume was measured from the weight of the strip before and after sampling.

# **Comment 3**

**Intra- and inter-assay variation could also be an important parameter to assess. I would suggest authors to perform precision sampling and sample retention test experiments on (1) same lot of microfluidic paper and same operator over a short period of time (intra-assay) and (2) different lot over an extended period of time performed by multiple operators (inter-assay).**

**Answer**

We would like to thank the reviewer for the comment in terms of precision. For intra-assay, in this revision, we conducted an extra experiment to evaluate the SMP (d=75, p=150 µm) selected based on these tests (Figure 7&8) in the comparison with filter paper. On this experiment, we conducted liquid sampling for the two types of papers (SMP and filter paper) 10 times with a new paper strip every time. To avoid the variation originating from human error, sampling (that is pulling the paper strip out of liquid) was conducted with machine-controlled set-up in precision sampling tests. Please check the results of the newly added section from the line 250 of the page 10 in the revised manuscript.

It is doubtful whether inter-assay in the current stage makes any important sense. It is because the polymer formulation of SMP should be reconsidered probably before actual implementation into a diagnostic system and long-term and multi-person tests should be after that. The subject of this work is to illustrate the usability of SMP in the field of measurement using paper strip and, hence, we did not conduct the inter-assay. However, this point is very important for actual implementation of diagnostic system so we added discussion related with the inter-assay from the line 270 of the page 11 in the revised manuscript.

***The added section in the line 250 of the page 10 (line 270 appear in the last paragraph):***

**Comparison between synthetic microfluidic paper and filter paper**

Fig 9 (a) shows the coefficient of variation (CV) of sampling volume for the SMP and the filter paper, which was calculated as the ratio of the standard deviation to the mean value (N = 10). The actual volume sampled by each paper is shown in SI Fig S2. The CV value of the SMP was slightly greater than that of the filter paper. However, we performed F-test on the two normalized data set (ratio of sampled mass over its mean mass) of the SMP and the filter paper by “ftest” function in Excel, and, when an α-level of 0.05 was used, no significant difference was found between them (P = 0.382 > α). Fig 9 (b) shows the R_F. The error bars represent standard deviation. T-test performed on the two groups by “ttest” function in Excel showed no significant difference between the SMP and the filter paper (P=0.078 > α), when an α-level of 0.05 was used. On the other hand, F-test performed on the two groups showed that there is a significant difference between variation for the two samples (P = 0.0056 < α), when an α-level of 0.05 was used.

In summary of the comparison between the SMP and the filter paper, they were found to have comparable intra-assay precision in water sampling and retention, but robustness to mechanical disturbances was shown more in the SMP than the filter paper. However, there is a big difference between the SMP and the filter paper: the former was fabricated manually in a laboratory but the latter was done by mass-production process. Actually, the mass variation of the SMP and the filter paper was 0.078 and 0.033 in CV. Automated process or reduction of manual process, if possible, are expected to decrease variation of mass in SMP and consequently variation of sampling volume.

From a future perspective, sampling liquid by SMP has challenges in terms of evaluation for long-term or multi-person use. In this work, the usability of SMP for precise sampling has been confirmed in short-term period. It is also important to know long-term capability to perform precise sampling as a reference for a shelf-life. To get longer-term capability, optimization of polymer formulation for the SMP is one of the issues to be considered. Another challenge to be addressed is multi-operator tests. Depending on the application destination, it is necessary to test with a wide range of subjects who have different age and presence or absence of disease.

# **Comment 4**

**For the precision sample experiment, in addition to the coefficient of variation, it would be interesting to also show the actual volume collected by each strip/filter paper (by including errors bars and calculating difference between each method).**

**Answer**

We newly formatted electronic supporting information Fig S1 including the actual measured values.

**Fig S1. Mass per unit volume of water sampled by synthetic microfluidic papers in precision sampling experiments.**

# **Comment 5**

**Line 195, please discuss/provide an explanation on why the synthetic microfluidic paper with (d, p) of (75, 150), (75, 225), and (100, 300) was more suitable for producing consistent sampling volume than others.**

**Answer**

Our goal in this experiment section is to find synthetic microfluidic paper condition with small variation in sampling volume. It is same meaning with finding geometric condition of synthetic microfluidic paper with small CV. With this regard, these three conditions are better than other tested conditions for sampling. We slightly modified a sentence in the line 222 of the page 8 in the revised manuscript.

***The sentence in the previous manuscript:***

Among the conditions tested, immersion method I or II using the synthetic microfluidic paper with (d, p) of (75, 150), (75, 225), and (100, 300) was suitable for producing consistent sampling volume.

***The modified sentence in the line 222 of the page 8 in the revised manuscript:***

Among the conditions tested, immersion method I or II using the SMP with (d, p) of (75, 150), (75, 225), and (100, 300) seemed better for sampling liquid when one needs small CV (< 0.05).

# **Comment 6**

**Line 206-208, “the values of the synthetic microfluidic papers with a pitch rate of 1.5 and 2.0 and (d, p) of (75, 112.5), (75, 150), (100, 150), and (100, 200) were approximately the same as that of filter paper” and “the synthetic microfluidic paper with a pitch ratio of 3.0 had a greater value”. Is it significant? Authors should calculate significance of difference between strip themselves and filter.**

**Answer**

We added a Table S1 to show the P values as the results of T-test on each pair of SMP. As shown in this table, there is significant difference between these parameters ((d, p) of (75, 112.5), (75, 150), (100, 150), and (100, 200)) and the others, when an α-level of 0.05 was used.

As the answer for comment 3, we considered the previous data on the precision sampling tests and the sample retention tests (Figure 7&8) as the experiments for the selection of SMP conditions and the sampling method, and we conducted an extra experiment to evaluate the SMP (d=75, p=150 µm) in the comparison with filter paper. Here, we discussed whether there is a significant difference in variation for precision sampling tests and sample retention test based on T-test and F-test. Please see the new results from the line 250 of the page 10 in the revised manuscript (same as the answer for comment 3).

**Table S1 P-value derived from T-test on two types of synthetic microfluidic paper**

# **Comment 7**

**Time between collection and analysis is also a parameter that can widely change (especially when performed directly by patient) and induce important variation. Therefore, authors should specify the period of time between sample collection and measurements they performed. In addition, I would also suggest authors to evaluate potassium concentration at different time after collection in order to assess if microfluidic strip can result in better sample preservation than filter paper.**

**Answer**

In this experiment, the concentration was measured soon after liquid sampling (<10 s). Similarly, when used by patients, it is assumed that the concentration is measured soon after sampling. To achieve the rapid performance of sampling and measurement, we keep the measurement system as simple as possible, *i.e.* combination of a paper strip and a portable densitometer. Therefore, in this paper, we did not focus on time interval between sampling and concentration measurement.

# **Comment 8**

**Authors assessed the interest of their device in a context of “urinalysis” for a patient of implantable artificial kidney. Although it is surprising that authors do not use urine sample being given the easy access, they claimed that they used water to test their device. What is the pH of this solution? pH can impact ion measurement and it would have been a minimum to use a solution with a pH equal to urine (~6). Even more interesting, potassium measurement could have been tested with solution with different pH (4-8).**

**Answer**

First, pH of the used solution is approximately 7. In our proposed system, we are assured that aqueous potassium chloride solution was sufficient for use as a model of urine. To convince the reviewer of the appropriateness, we should have explained in more detail the difference of the urine of a patient of implantable kidney from ordinary urine. The implantable artificial kidney developed by our group has a mechanism whereby the filtrate generated by hemofiltration is conveyed to the bladder, turning into urine. Our previous studies showed that this filtrate is equivalent to blood plasma with respect to waste and electrolytes (See also N. To, *et al.*, *Front. Bioeng. Biotechnol.* 3 (2015) 70). Since the blood plasma is mostly composed of water and includes the electrolyte component about 1% and its pH does not deviate significantly from 7, we determined using an aqueous potassium chloride solution as a model urine for artificial kidney. Perhaps, our previous text reminds readers of a general urinary testing at this stage so we added several sentences to state that our urinary measurement was in a specific case (the line 186 of the page 7).

***The added sentence in the line 186 of the page 8:***

To see if the SMP can be used for measurement system using a paper strip, we conducted potassium concentration measurement using SMP, supposing urinalysis for a patient of implantable artificial kidney. The implantable artificial kidney being developed by our group has a mechanism whereby the filtrate generated by hemofiltration is conveyed to the bladder, turning into urine [21]. Our previous studies showed that this filtrate is equivalent to blood plasma with respect to waste and electrolytes [21]. Since the blood plasma is mostly composed of water and includes the electrolyte component about 1% and its pH does not deviate significantly from 7, we determined using an aqueous potassium chloride solution as a model urine for potassium measurement in this study.

# **Comment 9**

**Line 237, authors claimed “This result suggests that the synthetic microfluidic papers are applicable for precision sampling of urine for the potassium concentration measurement.” This seems overstated since authors did not assess potassium concentration in urine. In addition, sample nature (water spiked with K+?) needs to be specified in Materials and methods section.**

**Answer**

We agree the reviewer’s point against our claim. We understand that our results were not enough to show applicability for actual urine measurement for broad diagnostics. Thus, we modified the sentence in the line 299 of the page 10 as follows: “This result suggests that the SMP is applicable to precision sampling of aqueous solution in the potassium concentration measurement.” Preparation procedure of the K^+^ aqueous solution was specified in the line 205 of the page 8.

***The sentence in the previous manuscript:***

This result suggests that the synthetic microfluidic papers are applicable for precision sampling of urine for the potassium concentration measurement.

***The modified sentence in the line 299 of the page 10 in the revised manuscript:***

This result suggests that the SMP is applicable to precision sampling of filtrate of a patient of implantable artificial kidney, followed by the potassium concentration measurement.

***The added sentence in the line 205 of the page 8:***

The sample solutions contained potassium ion concentrations of 60, 140, 200, and 360 ppm, which were prepared by dissolving 60, 140, 200, and 360 mg of potassium chloride in 1 L of ultra-purified water, respectively.

# **Comment 10**

**Sensitivity could also have been evaluated by measuring the lowest achievable potassium concentration with the different methods.**

**Answer**

We appreciate the reviewer’s point about sensitivity. We think that knowing the lowest achievable concentration is not absolutely necessary in our specific case.

The human potassium standard range is 140 ppm to 200 ppm (3.6 mEq/L to 5.0 mEq/L). To detect an abnormality of potassium concentration in a patient of artificial kidney, it is necessary to measure a concentration deviating from the standard range up to 10 ppm. In this context, the range of 60 ppm to 360 ppm (the tested range) would be enough. In addition, a resolution capable of distinguishing orders of 10 ppt is sufficient, on which our system meets the case.

Sensitivity is heavily dependent on densitometer, which is not our focus in the present paper.

Reviewer 2 -Minor point-

# **Comment 11**

**In introduction (line 52), please specify what “unprecise sampling” means (volume variation, stability, concentration variation, etc.?)**

**Answer**

We would like to thank this comment. In the context, the “unprecise sampling” was volume variation. To avoid misunderstanding, we replaced “unprecise sampling” with “volume variation” in the line 55 of the page 3.

# **Comment 12**

**In introduction (line 66), please remove “or urine”**

**Answer**

We deleted the word (“or urine”) out of the line 67 of the page 3.

# **Comment 13**

**In “fabrication process” in Materials and methods section, authors claimed that “The synthetic microfluidic paper … is more robust against collapse than the structures containing vertical straight pillars”. Reference (or data) should be included to support this affirmation.**

**Answer**

We have added reference of the following paper (listed in the previous reference list) from the line 100 of the page 4.

[19] Hansson J, Yasuga H, Haraldsson T, van der Wijngaart W. Synthetic microfluidic paper: high surface area and high porosity polymer micropillar arrays. Lab Chip. 2016;16: 298–304. doi:10.1039/C5LC01318F

# **Comment 14**

**Please specify how CV has been calculated**

**Answer**

We have added how we obtained the CV from the line 217 of the page 9.

***The added sentence in the line 217 of the page 9:***

Fig 7 shows the coefficient of variation (CV) of sampling volume for each strip, which was calculated as the ratio of the standard deviation to the mean value (n = 5). The actual volume sampled by each paper is shown in Supporting Information (SI) Fig S1.
